# Measuring Depression in a Non-Western War-Affected Displaced Population: Measurement Equivalence of the Beck Depression Inventory

**DOI:** 10.3389/fpsyg.2017.01670

**Published:** 2017-09-26

**Authors:** Nuwan Jayawickreme, Jay Verkuilen, Eranda Jayawickreme, Kaylaliz Acosta, Edna B. Foa

**Affiliations:** ^1^Department of Psychology, Manhattan College, New York City, NY, United States; ^2^Department of Educational Psychology, City University of New York, New York City, NY, United States; ^3^Department of Psychology, Wake Forest University, Winston-Salem, NC, United States; ^4^Department of Psychiatry, Center for the Treatment and Study of Anxiety, University of Pennsylvania, Philadelphia, PA, United States

**Keywords:** depression, war survivors, Sri Lanka, Beck Depression inventory, measurement equivalence

## Abstract

Depression is commonly seen in survivors of conflict and disaster across the world. There is a dearth of research on the validity of commonly used measures of depression in these populations. Measurements of depression that are used in multiple contexts need to meet *measurement equivalence*, i.e., the instrument measures the same construct in the same manner across different groups. The Beck Depression Inventory (BDI) was administered to female trauma survivors in the United States (*n* = 268) and female survivors of war in Sri Lanka (*n* = 149). Three metrics of measurement equivalence—structural, metric, and scalar—were examined. Two- and three-factor structures of the BDI that have been identified in other populations did not provide a good fit for our data. However, a bifactor model revealed a similar general distress dimension across populations, but dissimilar secondary dimensions or subfactors. The Sri Lankan subfactor comprised of predominantly somatic symptoms and the United States subfactor comprised of cognitive and somatic symptoms. While intercepts of individual BDI items differed, their differences seem to be offsetting. Total BDI scores across these two populations are roughly comparable, although caution is recommended when interpreting them. Making comparisons on subscales is not recommended.

## Introduction

It is estimated that around 65.3 million individuals—the vast majority from non-Western, low-income countries—are currently displaced by violent conflict and humanitarian disasters, including 21.3 million refugees and 40.8 million internally displaced individuals (United Nations High Commissioner for Refugees, 2016). It has been well-established that due to exposure to multiple chronic stressors, this population has high levels of mental illness (Fazel et al., 2005). One of the more widely prevalent and widely studied mental illnesses in this population is depression (Steel et al., 2009), which is a significant contributor to the global burden of disease (Whiteford et al., 2015). Governments, as well as international and local agencies servicing survivors of conflict and disaster, often have limited resources for mental health services (Saxena et al., 2007) and thus, it is important that cross-culturally valid instruments measuring depression and other mental illnesses exist so that survivors who have clinically significant depression and other mental illness can be identified.

With regards to the assessment of depression, the Beck Depression Inventory (Beck and Steer, 1987) is one of most commonly used self-report instruments for assessing the severity of depression worldwide (Richter et al., 1998; Shafer, 2006). The ease with which the BDI can be administered makes it an attractive measure to efficiently and economically determine levels of depressive symptomology in populations of interest. Despite the development of the revised BDI-II 20 years ago (BDI-II; Beck et al., 1996), the original BDI is still widely used in clinical and research settings in both North America (Foa et al., 2005; Gillespie et al., 2009; Veerman et al., 2009; Delisle et al., 2012; Jayawickreme et al., 2014; Udo et al., 2015) and Europe (e.g., Suija et al., 2012; Lahlou-Laforet et al., 2015; Wardenaar et al., 2015). Strikingly, the BDI continues to be widely used to assess for depression in refugees and internally displaced and war-impacted populations (Mghir and Raskin, 1999; Bhui et al., 2003; Ghazinour et al., 2003; Yurtbay et al., 2003; Basishvili et al., 2012; Luitel et al., 2013; Kuittinen et al., 2014).

However, despite the wide use of the BDI and other measures developed for Western clinical populations in non-Western, post-disaster settings, there is a dearth of studies examining the cross-cultural validity of these measures (Van Ommeren, 2003; Bass et al., 2007). In a review of 183 research studies focusing on refugee populations, Hollifield et al. (2002) found that half of the studies did not provide any evidence of validity and reliability for the instruments used. Given that there is now considerable evidence for cross-cultural differences in both the experience and presentation of depression (e.g., Paniagua, 2000; Karasz, 2005; Ryder et al., 2008), it cannot be assumed that just because an instrument is valid and reliable in one context, it will be so in other contexts. Furthermore, one cannot assume that scores on a particular instrument are meaningful in the same way across cultural settings. This point is especially relevant to those providing services to displaced and war-affected populations, who often rely on instrument clinical cutoff scores to identify those who are mentally ill. For example, BDI cutoff scores have been used to determine depression severity in war-affected Lebanese individuals (Farhood and Dimassi, 2012), war-affected Iraqis (Magruder et al., 2015), and Somali refugees (Mölsä et al., 2014). Furthermore, researchers and policy makers use these cutoff scores to compare the relative rates of mental illness across different countries (e.g., Van Hemert et al., 2002).

Before comparisons of scores between different cultural groups can be made, one must first ensure that the instrument in question meets *measurement equivalence* (also referred to as *measurement invariance* in the literature); in other words, the instrument must measure the same construct in the same manner across different cultural contexts (Chen, 2008; Millsap, 2011). Measurement equivalence is established by meeting four increasing restrictive criteria: (1) functional equivalence; (2) structural equivalence; (3) metric equivalence; and (4) scalar (or full score) equivalence (Fischer and Fontaine, 2011; Millsap, 2011)^1^. *Functional equivalence* is established when the same construct (e.g., depression) can be observed across different groups. Functional equivalence is typically established through a combination of ethnographic research, case studies, and epidemiological surveys (Lopez and Guarnaccia, 2000). There is now a large body of research indicating that depression is indeed seen globally (Kessler and Bromet, 2013) and there is some evidence (e.g., Jayawickreme N. et al., 2012; Haroz et al., 2016) that measures of depression developed in the Western world at least partially captures the experience of depression in low and middle-income countries in the global south.

*Structural equivalence* is established when the factor structure of the measure is the same across different groups. With regards to the BDI, Beck and Steer (1993) note that the measure can be divided into two factors, a subscale of cognitive and affective symptoms consisting of the first 13 items, and a subscale of somatic and performance-affecting symptoms consisting of the last eight items (see Table 1). This factor structure has received considerable factor analytic support (Steer et al., 1992; Beck and Steer, 1993). Another factor structure, comprising of three factors, Negative Attitudes toward the Self, Performance Impairment, and Somatic Disturbances, has also been identified in numerous samples (Beck et al., 1988; see Table 1). A meta-analysis of factor-analyses for the BDI (total number of individuals = 13,643, total number of studies = 33) found that this three-factor solution provided the best fit (Shafer, 2006). It should be noted, however, that these factor solutions have been tested predominantly in European-American, middle class samples in the United States (Shafer, 2006).

**Table 1:** Item mapping for confirmatory factor analysis—the Beck Depression Inventory (Beck and Steer, 1987).

**Item**	**2-Factor model (Beck and Steer, 1993)**	**3-Factor model (Beck et al., 1988; Shafer, 2006)**
1. Sadness	C-A	NS
2. Pessimism	C-A	NS
3. Past failure	C-A	NS
4. Loss of pleasure	C-A	NS
5. Feelings of guilt	C-A	NS
6. Feelings of being punished	C-A	NS
7. Self-dislike	C-A	NS
8. Self-criticalness	C-A	NS
9. Suicidal thoughts/wishes	C-A	NS
10. Crying	C-A	NS
11. Irritability	C-A	PI
12. Loss of Interest	C-A	PI
13. Indecisiveness	C-A	PI
14. Lack of attractiveness	P-S	NS
15. Loss of energy	P-S	PI
16. Changes in sleeping pattern	P-S	SS
17. Tiredness or fatigue	P-S	PI
18. Changes in appetite	P-S	SS
19. Weight loss	P-S	SS
20. Health concerns	P-S	PI
21. Loss of interest in sex	P-S	PI

*C-A, Cognitive-affective factor; P-S, Performance-somatic factor; NS, Negative attitudes about the self; PI, Performance impairment; SS, Somatic symptoms*.

The heterogeneity of factor models for depression measures, including the BDI, has led some to argue that bifactor factor models better account for the nature of depression (Ward, 2006; Brouwer et al., 2013). Bifactor models have a single general factor that consists of all items in the measure, and subfactors consisting of a subset of items that reflect a narrower construct. As such, these models identify a general factor and any subfactors that account for variance over and above the general factor (Dere et al., 2015). de Miranda Azevedo et al. (2016) tested a bifactor model for the BDI using data from 13,100 patients with myocardial infarction from five countries. The best fit was found for a model that had a general factor that included all 21 items in the measure, and two subfactors, a somatic/affective subfactor and a cognitive/affective subfactor.

The third level of measurement equivalence is *metric equivalence*. A measure has metric equivalence when individual items load onto factors with the same strength across different populations. This type of variance allows one to make indirect comparisons between these populations, for example, whether predictive relationships found in one population can be found in the other or if changes in symptoms across time are similar across populations. However, in order to directly compare total scores across multiple populations, the fourth and last level of measurement equivalence—*scalar equivalence*—has to be met. If a measure has scalar equivalence, then the same score on the measure refers to the same position on the underlying latent variable across populations. Scalar equivalence is usually determined by examining whether individual items have the same point of origin, or intercept in the groups being compared. Failing to establish metric and scalar equivalence for a particular measure can result in both the identification of false group differences and the inability to detect true group differences (Chen, 2008).

Two studies have looked at the measurement equivalence of the BDI across different countries. Azocar et al. (2001) examined measurement equivalence in the BDI in medical patients who were either Spanish-speaking Latinos or English-speaking U.S. nationals using differential item functioning (DIF) analysis; they found that Latinos were more likely to endorse crying and punishment and less likely to endorse inability to work compared to English-speaking U.S. nationals with the same total BDI score, indicating a lack of measurement equivalence. Nuevo et al. (2009) examined the measurement equivalence of the BDI across five European countries—UK, Ireland, Spain, Norway, and Finland—and found that while the measure met criteria for structural equivalence, only *eight* of the 21 items displayed no DIF, thus indicating a lack of measurement equivalence.

## The current study

No studies have yet examined measurement equivalence of the BDI in non-western, displaced populations, despite its wide use in those contexts. The current study focuses on females from such a population; female refugees and survivors of war have been found to have higher rates of depression (Ai, 2004; Seglem et al., 2011) and more severely impaired global functioning (Song et al., 2015) compared to men. Specifically, we investigate whether the BDI has measurement equivalence in internally displaced female survivors of the Sri Lankan civil war, which lasted from 1983 to 2009 (Jayawickreme et al., 2010). It is estimated that there are currently almost 45,000 internally displaced individuals in Sri Lanka (United Nations High Commissioner for Refugees, 2016). Similar to other displaced populations, Sri Lankan war survivors have endured multiple traumas and have high rates of mental illness, including depression (Jayawickreme N. et al., 2012). A survey of 1,517 households in the Jaffna district in Sri Lanka—an area that was most severely impacted by the civil war—found that 22% of individuals had a diagnosis of depression, with displaced individuals more likely to report symptoms of depression compared to long-term residents (Husain et al., 2011).

Identifying an equivalent Western sample that one can compare a non-Western war-affected population is challenging, given that experiences that non-Western war survivors undergo—multiple traumas, destruction of community, lack of security—are uncommon in the Western world. In order to have a degree of equivalence of life experience between the two groups, we used a sample of U.S. females from a major city—Philadelphia, PA—with a history of trauma. While the trauma suffered in a war context is more chronic compared to those suffered in Western settings, Western urban contexts are nevertheless associated with high levels of trauma exposure (e.g., Gillespie et al., 2009; Khoury et al., 2010; Brown and Mellman, 2014; Whitbeck et al., 2015). Indeed many other risk factors for PTSD besides trauma exposure are concentrated in Western urban contexts, such as risk of future victimization, fewer resources, poor coping skills, and multiple daily stressors (Kelly et al., 2010).

In the current study, we examined the three types of measurement equivalence that can be established through statistical analyses of responses on a measure—structural, metric, and scalar equivalence. We aimed to first identify an optimal factor structure for the BDI in war-affected Sri Lankan females and U.S. females with a history of trauma. To do this, we tested a series of potential baseline factor models. Following this, we examined the measurement equivalence of the BDI in these two culturally distinct groups.

## Methods

### Participants

The US sample consisted of 268 female survivors of sexual or non-sexual abuse who were recruited by the University of Pennsylvania's Center for the Treatment and Study of Anxiety through newspaper advertisements and police and hospital referrals as part of a prospective assessment study. Participants were assessed at <4 weeks after the assault. Exclusion criteria for this study included a previous diagnosis or current presence of organic mental disorder, schizophrenia, or paranoid disorder as defined by the DSM-III-R (American Psychiatric Association, 1987), or if they were illiterate in English. Furthermore, participants who were involved in an ongoing intimate relationship with their perpetrator were excluded.

The Sri Lanka sample consisted of 149 female Tamil survivors of that country's civil war who received psychosocial assistance from the Family Rehabilitation Center (FRC), a nongovernmental organization based in Sri Lanka. Data were collected from FRC clinics in Jaffna, Batticaloa, Trincomalee, Vavuniya, and Nallur, all urban centers that were greatly impacted by the civil war. Participation in the study was voluntary and participants were paid 100 Sri Lanka rupees (i.e., enough to purchase lunch or dinner, ~75 U.S. Cents).

All procedures were approved by the Institutional Review Board at the University of Pennsylvania, Philadelphia, PA, United States, and by the Ethics Committee at the University of Peradeniya, Sri Lanka.

### Measures

The following measure was administered to participants in both samples as part of a larger battery:

*The Beck Depresson Inventory* (BDI; Beck and Steer, 1987) is a 21-item self-report inventory that requires respondents to rate the severity of their depression symptoms over the previous 2 weeks. Each item consists of a graded series of four alternatives that range from 0 to 3 in terms of severity. Total scores on the BDI range from 0 to 63. The original English language version of the inventory was administered to the United States sample and a Tamil translation was administered to the Sri Lankan sample. BDI scores are reliable and valid indicators of depression in Western samples (Killgore, 1999) and correlate with clinical ratings and other measurements of depression (Beck et al., 1988). Beck et al. (1988) reviewed the literature on the psychometric properties of the BDI and found mean coefficients alpha of 0.86 for psychiatric patients and 0.81 for nonpsychiatric patients. Furthermore, Hollifield et al. (2002) found that the BDI met all five criteria used to evaluate the suitability of the instrument for use in non-Western refugee populations, namely purpose, construct definition, design, developmental process, and reliability and validity.

A rigorous translation process was employed to obtain an equivalent Tamil language translation of the BDI for use in the Sri Lankan sample and is described in detail by Jayawickreme N. et al. (2012). Briefly, the BDI was first translated into Tamil by two physicians who were both native speakers of Tamil and fluent in English. These physicians used the translation monitoring form (Van Ommeren et al., 1999) to record the translation, lexical back translation, and evaluation of each item. This translation was then discussed in a focus group of three Tamil men and three Tamil women educated to a secondary school level in order to ensure face validity of the measure. After corrections were made, the BDI was back-translated to English by a second pair of bilingual physicians. The back-translated BDI was reviewed by Robert J. DeRubeis, Ph.D. and four graduate students from the University of Pennsylvania to ensure semantic equivalence. This back-translation was then translated back to Tamil by a third pair of bilingual physicians and then piloted in a group of three Tamil men and three Tamil women who were educated to the secondary school level. Pilot testing using focus groups suggested that the BDI could be easily understood in a Tamil-speaking population (for more details, see Jayawickreme E. et al., 2012).

#### Trauma exposure

Trauma exposure was assessed in the Sri Lankan sample using the Trauma Exposure sub-section of *the Penn/RESIST/Peradeniya War Problems Questionnaire* (PRPWPQ; Jayawickreme et al., 2009). The PRPWPQ was developed specifically to assess war problems in Sri Lanka (see Jayawickreme et al., 2009). In the Trauma Exposure section of the PRPWPQ, participants indicate whether they have experienced the trauma in question, and if so, the number of times they had experienced that trauma. Trauma exposure in the U.S. sample was assessed using the *Standardized Assault Interview* (SAI; Rothbaum et al., 1992). The SAI is a 136-item semi-structured interview that includes questions on assault characteristics such as injury and life threat.

#### Demographics

Age and ethnicity were assessed as part of the SAI in the U.S. sample. In the Sri Lankan sample, participants completed a separate demographics form in which they indicated their age and ethnicity.

### Data analytic strategies

#### Missing data

There was a modest amount of missing data. The US respondents had no missing data, whereas the Sri Lankan respondents had a small amount of missing-at-random data, <3%, with most variables being fully observed. Because of this, we used full information estimation using maximum likelihood (ML) and weighted least squares missing values (WLSMV) in Mplus. These methods generate consistent estimates in the presence of partially observed responses.

#### Measurement equivalence

Our strategy for establishing measurement equivalence, in line with that outlined in McDonald (1999), was to start with the two-factor (2FCFA) and three-factors (3FCFA) confirmatory factor (CFA) models that have been used in the literature previously. McDonald's approach emphasizes fitting CFA models that are theoretically motivated, with follow-up analysis to consider model revision. For this, we used exploratory bifactor analysis (Reise, 2012; Mansolf and Reise, 2016). We used Mplus Version 7.4 (Muthén and Muthén, 1998–2015) to fit the factor analytic models to these data.

As noted earlier, prior analysis with the BDI has predominately focused on two-factor (e.g., Beck and Steer, 1993) and three-factor models (e.g., Shafer, 2006). We refer to these as 2F and 3F, respectively. The 2F model has items 1 through 13 loading on the Cognitive and Affective factor and items 14 through 21 loading on the Performance-Somatic factor. The 3F model has items 1 through 10 and 14 loading on the Negative Attitudes toward the Self factor, items 11, 12, 13, 15, 17, 20, and 21 on the Performance Impairment factor, and items 16, 18, and 19 on the Somatic Disturbances factor (see Table 1).

The usual approach to measurement equivalence focuses on a set of increasingly constrained multi-group models. However, in our analyses, we employed a useful, new method proposed by Asparouhov and Muthén (2014) known as *alignment*, which attempts to provide an answer to the question of whether factor structure is approximately invariant. Instead of fitting a sequence of increasingly strict constrained CFAs, the alignment method fits configural models (i.e., same pattern of loadings on factors but with freely varying thresholds, loadings, factor variances, and covariances) to each group and then uses a method similar to Procrustes rotation to put the different solutions in maximum possible alignment with each other. It then provides adjusted hypothesis tests for parameter equality, corrected for multiple comparisons, which help determine whether the groups are in alignment. This method requires estimation by ML. Prior research suggests that failing to correct for ordinality has deleterious consequences for estimates (Li, 2016). Because the items are ordinal, we used robust ML with Gaussian quadrature. This is important because some items were notably skewed, particularly in the USA group, where a number of items had modes at the floor. For all models, we used 50 random starts followed by 20 fits of each model to completion to assure that the models did not converge to local optima.

After examining these CFA models, we used exploratory bifactor analysis to analyze the data, also considering two-factor and three-factor models, denoted 2FEFA and 3FEFA, respectively. Because Mplus does not provide a full set of fit statistics (Root Mean Squared Error of Approximation, RMSEA; Close Fit Index, CFI; Tucker-Lewis Index, TLI; Standardized Root Mean Residual, SRMR; Weighted Root Mean Residual, WRMR) for model comparison under ML, we re-estimated these models using WLSMV, which does. The resulting model estimates were essentially the same. While Kline (2016) notes that there are no perfectly accepted reference values for fit statistics, RMSEA ≤ 0.05, CFI/TFI ≥ 0.95, SRMR ≤ 0.05, and WRMR ≤ 1, are commonly used. We use these for our reference values. Kline (2016) also recommends examination of covariance residuals, which we also do.

The logic of the bifactor model is to seek a primary factor and consider systematic variation past that captured by secondary factors, with items having a loading on the general factor and one secondary factor (hence subfactor). In sub-fields such as educational psychology, the general factor might be overall ability while subsidiary factors represent specific domains of knowledge or sources of nuisance variation, such as item passage effects. In a clinical setting, the primary factor is typically distress, whereas specific factors represent groups of similar symptoms. To interpret the bifactor model, one considers the size of the loadings on the general factor compared to secondary factors. If an item's loading on the general factor is larger than on the subfactor, it is typically considered a better measure of the general factor whereas, vice versa, if it loads more strongly on the subfactor, it is a better measure of the secondary factor. DeMars (2013) provides helpful guidance on interpreting bifactor models.

Jennrich and Bentler (2011, 2012) proposed the bifactor rotation for exploratory factor analysis, which first fits an EFA for a specified dimension and then rotates toward a bifactor structure. Geomin is a recommended rotation method when cross-loadings are anticipated (Browne, 2001) and the oblique bi-geomin is an adaptation that seeks a bifactor structure while allowing the solution to be oblique.

While there are confirmatory bifactor models, we did not have a clear confirmatory structure for the bifactors as only a single study (de Miranda Azevedo et al., 2016) has examined bifactor models in the BDI; however, we expected the items to load on the general factor in a similar way. To assess the congruence between groups, we made use of Tucker's congruence coefficient (Fischer and Fontaine, 2011) to measure the congruence of the resulting exploratory bifactor solutions between the groups. Tucker's coefficient of congruence for a vector of loadings on a particular dimension in two groups over *k* items is

$$C\left(\lambda_{1}\lambda_{2}\right)=\frac{\sum \lambda_{1k}\lambda_{2k}}{\sqrt{\sum \lambda_{1k}^{2}\lambda_{2k}^{2}}}$$

Essentially Tucker congruence is an uncentered Pearson correlation coefficient, which is appropriate for congruence because, unlike Pearson correlation, it takes overall level into account. Guidelines proposed and tested in Lorenzo-Seva and ten Berge (2006) suggest that congruence values between loadings of 0.95 or higher are essentially viewed as the same, while congruence values <0.85 are viewed as poor.

## Results

### Descriptive statistics

Demographic information for both samples can be found in Table 2. There was a significant difference of age between the two samples, *t*_(254.94)_ = 10.58, *p* = 0.000. The average BDI total score was 15.87 (*SD* = 11.15) in the U.S. sample and 21.63 (*SD* = 11.23) in the Sri Lankan sample; this difference in means was significant, *t*_(401)_ = 4.99, *p* = 0.000. Item-level means and standard deviations for each sample can be found in Table 3.

**Table 2:** Demographic characteristics of participants.

United States sample (***n*** = 268)	
Age, Mean (*SD*)	30.7 (9.8)
Race	
African American, N (%)	181 (67.5)
European American, N (%)	76 (28.4)
Hispanic, N (%)	6 (2.2)
Other, N (%)	4 (1.5)
Unknown, N (%)	1 (0.4)
Sri Lanka sample (*n* = 149)	
Age, Mean (*SD*)	42.9 (12)
Race	
Tamil, N (%)	143 (96)
Unknown, N (%)	6 (4)

**Table 3:** Item-level means for the Beck Depression Inventory (Beck and Steer, 1987) in the United States (*n* = 268) and Sri Lanka (*n* = 149) samples.

**Item**	**United States Mean (*SD*)**	**Sri Lanka Mean (*SD*)**
1. Sadness	1.09 (0.97)	1.37 (0.88)
2. Pessimism	0.51 (0.84)	0.93 (0.88)
3. Past failure	0.59 (0.86)	1.01 (0.82)
4. Loss of pleasure	0.96 (0.92)	1.01 (0.90)
5. Feelings of guilt	0.53 (0.78)	0.74 (0.87)
6. Feelings of being punished	0.88 (1.23)	0.99 (1.11)
7. Self-dislike	0.54 (0.74)	0.89 (0.84)
8. Self-criticalness	0.72 (0.93)	0.80 (0.86)
9. Suicidal thoughts/wishes	0.24 (0.51)	0.60 (0.73)
10. Crying	1.00 (1)	0.93 (0.95)
11. Irritability	0.83 (0.74)	0.92 (0.83)
12. Loss of interest	0.73 (0.77)	0.88 (0.99)
13. Indecisiveness	0.77 (0.86)	1.17 (0.92)
14. Lack of attractiveness	0.69 (0.99)	0.91 (0.90)
15. Loss of energy	0.86 (0.85)	1.48 (0.99)
16. Changes in sleeping pattern	1.2 (1.10)	1.34 (0.96)
17. Tiredness or fatigue	0.93 (0.86)	1.08 (0.81)
18. Changes in appetite	0.84 (0.96)	1.16 (0.86)
19. Weight loss	0.40 (0.67)	0.94 (0.91)
20. Health concerns	0.58 (0.73)	1.15 (0.99)
21. Loss of interest in sex	0.88 (1.02)	1.32 (1.14)

*Possible range of scores on individual BDI items: 0–3*.

### Trauma exposure

Details on the levels of trauma exposure can be found for the U.S. sample in Table 4 and for the Sri Lanka sample in Table 5.

**Table 4:** Experienced Trauma in the United States Sample (*n* = 268) as assessed by the Standardized Trauma Interview.

	**Assault type**	
	**Rape/Sexual (%)**	**Non-Sexual (%)**
Assaulted by stranger	81 (60)	126 (88)
Under influence during assault	39 (29)	5 (4)
Assailant under influence	73 (55)	37 (26)
Verbally threatened	86 (64)	57 (40)
Weapon held to head/throat	49 (37)	36 (25)
Injuries (Type Tx Required)		
Minor (No Tx)	29 (22)	41 (29)
Minor (Outpatient Tx)	62 (46)	32 (23)
Major (Hospitalization)	6 (4)	7 (5)
Feared death or serious injury	100 (75)	92 (64)

**Table 5:** Trauma exposure in Sri Lankan sample (*n* = 149) as assessed by the Trauma Exposure subscale of the Penn/RESIST/Peradeniya War Problems Questionnaire.

**Respondents were asked if they had ever been…**.	**%**
**TORTURE**	
Beaten in detention	14.8
Subjected to electric shock while in detention	2
Tortured by being beaten with a bag containing petrol	1.3
Tortured by being pricked under the nail with a pin	2
Tortured by being burnt with a cigarette butt in detention	2.7
**EXPERIENCED/WITNESSED TRAUMA**	
Being raped	1.3
Being imprisoned	18.8
Being kidnapped	10.1
Caught in a land mine	2
Injured by air strikes or bomb explosions or sudden attacks	20.1
Witnessed the death of loved ones	50.3
Witnessed the injury of my loved ones	54.4
**NON-WITNESSED TRAUMA**	
Death of husband or wife due to the war	16.8
Death of child/children due to the war	16.1
Death of mother and/or father due to the war	11.4
Husband or wife been kidnapped	9.4
Children been kidnapped	14.1
Parents been kidnapped	3.4
Other family members been kidnapped	16.1
Children been handicapped	12.1
Husband/wife been handicapped	5.4

*As some Sri Lankan participants experienced more than one trauma, the percentages for the Sri Lankan sample do not add up to 100*.

### Measurement equivalence

The 2FCFA models both converged to regular solutions and the alignment method indicated that items 2 (pessimism), 3 (past failure), 9 (suicidal thoughts/wishes), and 16 (changes in sleeping pattern) differed in the thresholds for the Sri Lankan compared to the U.S. participants. Item 2's lowest threshold differed, with the Sri Lankan participants having a higher threshold. Item 3 reversed this pattern. On item 9, Sri Lankan participants had systematically higher thresholds, while on item 16 the pattern was reversed. In all, this pattern of differences among thresholds may in effect cancel out, at least insofar as the factors are fairly strongly identified by having many indicators, which would dilute the effect of difference.

In terms of alignment, while there were differences and thus we should be wary of assuming scalar equivalence, the groups were substantially aligned, with only a few items having differences in the threshold parameters. The latent mean differences of Sri Lankan participants compared to U.S. participants were 0.30 on factor CA (*SE* = 0.19, *p* = 0.125) and 0.60 on factor SP (*SE* = 0.159, *p* < 0.001). This suggests that the Sri Lankan participants had overall higher levels on both latent variables, with the one for Somatic-Performance being significantly higher.

The 3FCFA model ran for the USA sample and fit better than the 2FCFA. However, for the Sri Lankan participants it did not converge to a regular solution. In particular the factor correlations were extremely large. This was not unsurprising. The 3FCFA model had one factor that only had three items and was thus barely identified. As MacCallum et al. (1999) note, factor instability is particularly likely in a situation where one or more of the factors are not strongly identified by having many indicators, especially when the sample size is modest. The 3FCFA model's divergence for the Sri Lankan sample likely reflects that the 3FCFA model is questionable.

As mentioned previously MLR estimation in Mplus for ordinal items does not provide conventional fit statistics such as RMSEA, Tucker-Lewis Index, or the SRMR. However, following Bartholomew et al. (2008), Mplus provides tables of univariate and bivariate residuals, scaled on a z-score metric. Examination of these residuals showed that the 2FCFA model was reasonable on the USA sample but did not fit as well on the Sri Lankan sample. To provide conventional fit statistics we used WLSMV, which generates estimates very similar to MLR. These are in Table 6. Supplementary Table 1 (Sri Lanka) and Supplementary Table 2 (the U.S.) contain the estimated loadings of the CFA models as well as factor correlations.

**Table 6:** WLSMV Fit Indices for CFA and EFA Models of the Beck Depression Inventory (Beck and Steer, 1987).

**Model**	**Country**	**Chi square**	**df**	**RMSEA**	**CFI**	**TLI**	**WRMR**
2FCFA	Sri Lanka	354.541	188	0.08	0.92	0.92	1.03
	U.S.	366.82	188	0.06	0.96	0.96	0.99
3FCFA	Sri Lanka	−	–	–	–	–	–
	U.S.	331.37	186	0.05	0.97	0.96	0.93
2FEFA	Sri Lanka	316.26	169	0.08	0.93	0.92	0.93
	U.S.	246.72	169	0.04	0.98	0.98	0.05
3FEFA	Sri Lanka	233.79	150	0.06	0.96	0.95	0.06
	U.S.	194.59	150	0.03	0.99	0.99	0.04
4FEFA	Sri Lanka	195.97	132	0.06	0.97	0.95	0.05
	U.S.	159.14	132	0.03	0.99	0.99	0.03

### Exploratory bifactor analysis

For the U.S. and Sri Lanka participants, neither the 2FCFA or 3FCFA models fit well, thus failing to meet structural equivalence (and consequently failing to meet metric and scalar equivalence as well). The models did generally fit better for the U.S. sample. Furthermore, the 3FCFA model for the Sri Lankan participants did not converge to a regular solution. This led us to consider alternative models to determine if it is possible to find an adequately fitting model that makes substantive sense. One particular feature of the CFA analysis was the fact that the factors were very strongly correlated, which suggests that an overall general factor is likely to appear. Rather than attempting many essentially *ad hoc* modifications to the CFAs using correlated residuals or cross loadings, we preferred to consider exploratory factor analysis (EFA), following the advice of McDonald (1999), where CFA is first fit and EFA is fit to guide model revision.

EFA, despite the name “exploratory,” works best when guided by theoretically meaningful expectations, or working hypotheses. Our working hypothesis was that the loadings on the primary factor would be similar across groups but that there would be group differences in the constitution of the secondary factor or subfactor. In this sense, the primary distress factor represents universal aspects of depression whereas the secondary factor or subfactor represents cross-cultural differences in depression. We fit models for two, three, and four factors separately to each group, denoted 2FEFA, 3FEFA, and 4FEFA. We used WLSMV when fitting these models. All models converged to a regular solution without difficulty. In the USA data, the 2FEFA model appeared to be a reasonable fit. In the Sri Lanka data, the 2FEFA model was not as good a fit and the 3FEFA appeared better. The 4FEFA models were likely to overfit and as such, we did not consider them in detail. The fit statistics for these models can be found in Table 6 and loadings for the 2FEFA model are available in Table 7. The loadings for the 3FEFA and 4FEFA models can be found in Supplementary Table 3 (Sri Lanka) and Supplementary Table 4 (USA).

**Table 7:** Factor loadings for Exploratory Bifactor Model solution for the Beck Depression Inventory (Beck and Steer, 1987).

	**United States**		**Sri Lanka**	
	**Factor 1**	**Factor 2**	**Factor 1**	**Factor 2**
1. Sadness	**0.74**	−0.03	**0.71**	−0.05
2. Pessimism	**0.67**	−0.11	**0.58**	−0.25
3. Past failure	**0.78**	−0.25	**0.82**	−0.02
4. Loss of pleasure	**0.72**	0.05	**0.75**	0.00
5. Feelings of guilt	**0.62**	−**0.37**	**0.58**	−**0.42**
6. Feelings of being punished	**0.60**	−0.25	**0.46**	−0.07
7. Self-dislike	**0.74**	−**0.39**	**0.72**	−0.25
8. Self-criticalness	**0.72**	−**0.43**	**0.57**	−0.22
9. Suicidal thoughts/wishes	**0.76**	−0.17	**0.57**	−**0.35**
10. Crying	**0.55**	0.16	**0.59**	−0.10
11. Irritability	**0.53**	0.07	**0.64**	−**0.33**
12. Loss of Interest	**0.76**	0.13	**0.67**	−**0.32**
13. Indecisiveness	**0.78**	0.14	**0.75**	−0.04
14. Lack of attractiveness	**0.72**	−0.06	**0.63**	0.18
15. Loss of energy	**0.64**	0.20	**0.68**	0.20
16. Changes in sleeping pattern	**0.55**	0.16	**0.76**	**0.35**
17. Tiredness or fatigue	**0.66**	**0.33**	**0.63**	**0.38**
18. Changes in appetite	**0.60**	**0.40**	**0.63**	**0.44**
19. Weight loss	**0.55**	0.26	**0.51**	0.28
20. Health concerns	**0.65**	0.19	**0.48**	**0.42**
21. Loss of interest in sex	**0.66**	0.21	**0.55**	0.22

*Loadings 0.3 or greater are in bold*.

To compare the solutions, we use congruence indices. For the 2FEFA solution, the primary factor's congruence was 0.99, while for the 3FEFA solution the primary factor's congruence was 0.98. Essentially the two groups' overall distress factor's loadings were the same (However, we have good reason to suppose their intercepts are different, as discussed previously). The subfactors' congruence was poor, however. In the 2FEFA model, the second factor's congruence was 0.73 while for the 3FEFA models the second factor's congruence was −0.30 and the third factor's congruence was 0.59. However, these subfactors were not large compared to the primary dimension and contained many values near 0. This is consistent with our hypothesis that there would be a match on the primary distress factor but not on the subfactors.

**Figure 1:**
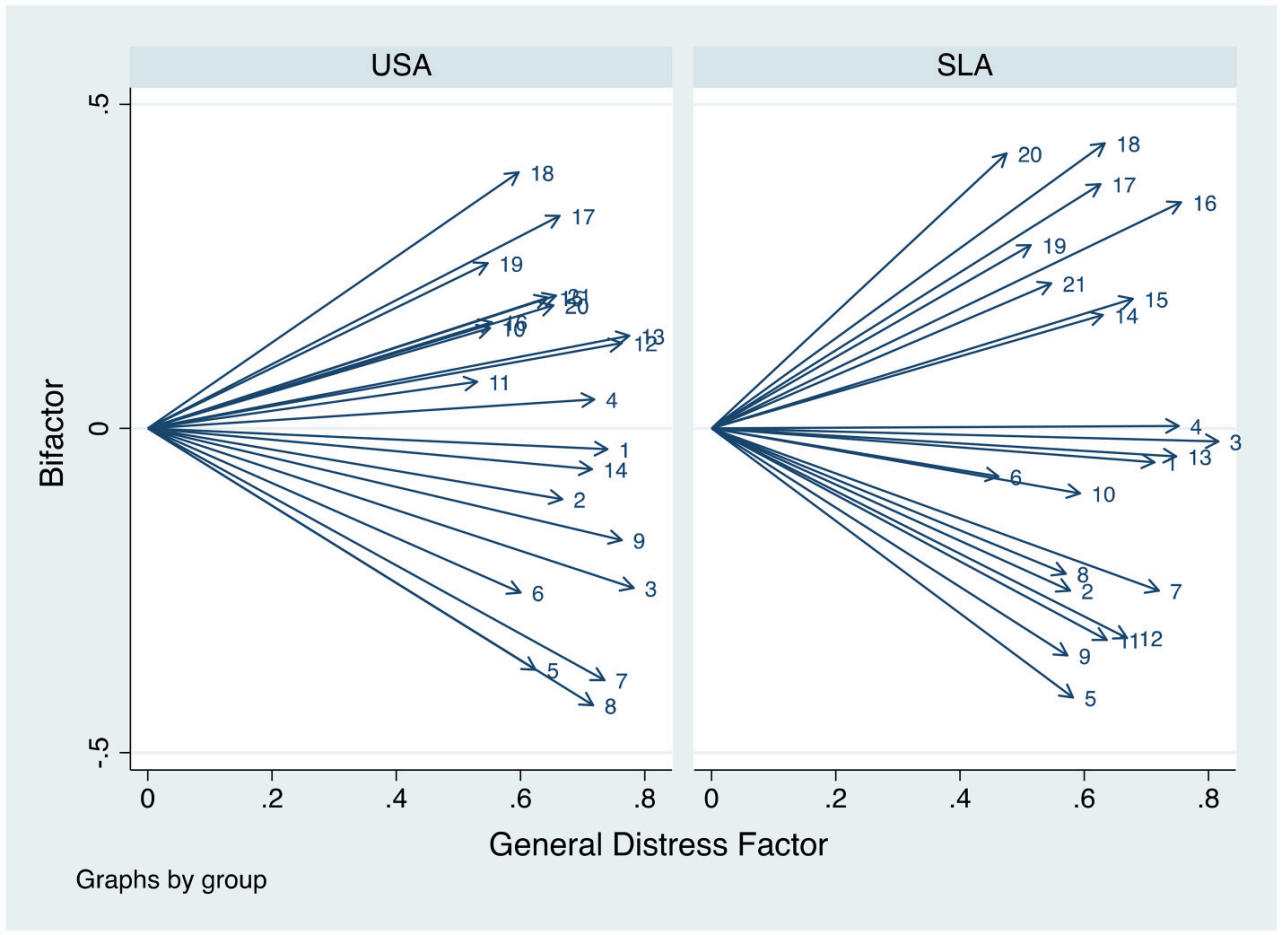
Standardized loadings for the general distress factors from the 2FEFA solution for the Beck Depression Inventory (Beck and Steer, 1987). USA, United States of America; SLA, Sri Lanka. Numbers (e.g., 1, 2, 3, etc.) indicate individual items on the BDI. See Table 1 for the specific item associated with each number.

To visualize this, Figure 1 shows the standardized loadings for the 2FEFA solution for the two groups. It appears to generate a “fan” structure, where some items appear to load positively and others negatively on the second dimension, while many have only small loadings on the secondary dimension. The 3FEFA model behaved similarly but was not so neatly visualized. The primary area of disagreement between the U.S. and Sri Lankan models involved the Performance-Somatic symptoms, which appear to load with greater frequency and with greater strength on the second dimension in the Sri Lankan model compared to the U.S. model (which has a combination of cognitive and somatic symptoms). In addition, Table 7 shows that the general factor loadings for both groups are always larger than the subfactors. Consistent with the results regarding congruence, the subfactors differ between groups. Finally, we calculated the factor analytic reliability for the general factors. For the USA sample these values are 0.90 for both the 2EFA and 3EFA models, respectively. The Sri Lanka sample's values are 0.89 for both the 2EFA and 3EFA models.

## Discussion

In order to use measures of mental illness that have been developed for Western settings in survivors of war living in non-Western contexts, it is essential that one first establish measurement equivalence for those measures. It is only when measurement equivalence is established that one can make comparisons between, and use similar cut-off scores across, populations. In the current study, we examined the measurement equivalence of the BDI, a self-report measure of depression that is widely used in non-western refugee and displaced populations, among a sample of Sri Lankan women who were displaced due to the recently concluded civil war. To our knowledge, this is the first comprehensive examination of the measurement equivalence of the BDI in a non-Western, war-affected, displaced population.

We failed to establish measurement equivalence for the BDI across females from Sri Lanka and the United States. Neither the two-factor or three-factor models found in previous studies provided an adequate fit, while a bi-factor model found a similar general distress factor in both the Sri Lankan and U.S. sample, but different secondary dimensions or subfactors. However, while the intercepts of individual items differed to some degree, their differences appear to effectively cancel off. This suggests that BDI total scores are, roughly speaking, comparable, although we recommend caution when interpreting them since respondents from the two countries may obtain the same total score through endorsing different items.

We do not recommend making comparisons on subscales (e.g., cognitive-affective vs. performance-somatic), as the subfactors identified by the bifactor model in the two samples were distinct from one another. The subfactor in the Sri Lankan sample was characterized by somatic symptoms (e.g., sleep changes, tiredness, appetite changes) whereas the subfactor in the U.S. sample consisted of both cognitive and somatic symptoms. These results suggest that somatic symptoms are a key element of the depression syndrome in Sri Lankan women, more so than in U.S. women. This distinction in depression symptoms between Sri Lankan and U.S. women mirrors the distinction in symptoms between Chinese and Canadian individuals identified by Ryder et al. (2008), who found that depression in Chinese individuals was characterized more by somatic symptoms compared to Canadian individuals, whose depression was characterized by both somatic and cognitive symptoms. Our finding is also consistent with prior research indicating that somatic symptoms are more prominently seen in refugee populations compared to non-displaced Western populations (Rohlof et al., 2014). This emphasis on somatization in non-Western populations may be due to the fact that in many non-Western cultures, the body and mind are believed to be integrated, leading to greater expression of mental distress through bodily experiences (Kirmayer and Young, 1998). This is in contrast to Western cultures, where beliefs about mind and body reflect the Descartian separation of the two and there is thus more of a distinction between psychological and physical experiences. Furthermore, non-Western cultures tend to emphasize emotional expression to a lesser degree compared to Western cultures, and to have a higher level of stigma attached to mental illness. Both these factors have also been hypothesized to lead to greater somatization of mental distress (Chentsova-Dutton and Tsai, 2002).

Several caveats should be noted. First, our findings could potentially be the result of sampling error. If we had a larger sample, we would have been able to split the sample and conduct comparisons. In particular, we would have been able to look at cross-validation, i.e., split the sample into two and run factor analysis on each of them to see if the loadings are stable and match each other.

Second, the two samples were different in terms of experienced trauma and age. Even though participants in both samples experienced trauma, the Sri Lankan women, unlike the U.S. women, were exposed to chronic trauma. This differential exposure to trauma in the two groups could underlie the findings of this study. Given that there was no variability in trauma exposure in the U.S. female sample—every participant reported experiencing a single trauma—we were unable to control for trauma exposure in our analyses. It is also possible that the observed differences between the U.S. and Sri Lankan samples could be due to the fact that the Sri Lankan sample was significantly older than the U.S. sample. No studies have examined the measurement equivalence of the BDI across age groups, but Missinne et al. (2014) found that another measure of depression, the eight-item version of the Centre for Epidemiological Studies Depression scale (Radloff, 1977), had measurement equivalence across two age groups (50–64 years of age; 65 years and older) in 11 different European countries. This suggests that age does not have an impact on how individuals respond on measures of depression.

Fourth, given that our U.S. sample was majority African-American, it is possible that that sample's responses on BDI reflect a pattern of symptomatology that is specific to that community. There are no studies examining whether African-Americans respond similarly to European-Americans on the BDI; however, the BDI-II (Beck et al., 1996), which has 17 of the 21 items on the original BDI, has been found to have equivalent responses patterns in African-American and European-American samples (Hambrick et al., 2010; Whisman et al., 2012). Given the similarity between the BDI and BDI-II, these findings suggest that the BDI may also have measurement equivalence in these two communities.

Fourth, the current study focused exclusively on females. There is evidence that there are gender differences in the symptom make up of depression in non-Western displaced populations (e.g., Renner and Salem, 2009); thus, the scope of the current findings is limited to females who have been displaced due to conflict.

Fifth, given the fact that all participants in this study experienced at least one traumatic event, it is likely that many participants had comorbid posttraumatic stress disorder (PTSD), whose symptoms overlap with depression (American Psychiatric Association, 2013). Thus, one could hypothesize that differences in symptom endorsement between Sri Lankan and U.S. females may in part be due to PTSD symptomatology. However, in a study of individuals with comorbid depression and PTSD, Post et al. (2011) found that while the two syndromes had overlapping symptoms, they were distinct empirical constructs. This suggests that the singular focus of the current study on a measure of depression is justified.

Despite these limitations, we believe that our study contributes to our understanding of how measures of depression developed in the Western world function in non-Western war-affected populations by comparing two groups that live in distinct cultural contexts: a Western, urban context and a non-Western, war-affected context. There is a paucity of this type of research, i.e., examining the measurement equivalence of commonly used measures of depression in war-affected contexts. Many researchers and practitioners assume that Western-developed measures such as the BDI have measurement equivalence across different contexts and thus use them to identify depression in non-Western war survivors; demonstrating that these measures actually function differently in those non-Western war contexts has real implications for the use of these measures in those situations. We posit that the current study makes a contribution that is of relevance to practitioners and researchers working with non-Western displaced populations and war survivors by providing evidence that the BDI functions differently in those settings and thus should be used with caution. Our findings indicate that while total scores on the BDI can, for the most part, be interpreted similarly across the two populations we sampled, the measure nevertheless failed to meet the strict requirements of measurement equivalence. Thus, researchers and practitioners who work with non-Western displaced populations cannot assume that just because a measure has been widely used or appears to tap into a construct seen on the ground, it can be used to identify those with clinically significant symptoms or to compare multiple groups. Lastly, our findings add to the growing body of literature indicating that bifactor models provide the best fit for the measurement of depression (e.g., Dere et al., 2015).

While there have been a handful of studies examining the measurement equivalence of popular measures of mental illness in culturally diverse (e.g., BDI-II; Dere et al., 2015) and refugee populations (e.g., Harvard Trauma Questionnaire; Rasmussen et al., 2015), more research is clearly needed to establish the psychometric properties of these measures. We do believe that future studies should attempt to identify specific variables that account for any lack of measurement equivalence of the BDI and other measures in non-Western war-affected contexts. Also, more research is needed to confirm the different response patterns to individual items of the BDI in the U.S. and Sri Lankan populations (as well as other populations impacted by war). In the meantime, caution must be used when interpreting and comparing scores from non-Western, war-affected, displaced populations.

## Ethics statement

This study was carried out in accordance with the recommendations of the Belmont Report, Institutional Review Board of the University of Pennsylvania with written informed consent from all subjects. All subjects gave written informed consent in accordance with the Declaration of Helsinki. The protocol was approved by the Institutional Review Board of the University of Pennsylvania.

## Author contributions

NJ conceived the research project, assisted with data collection, conducted data analysis, and wrote up the manuscript. JV conceived the research project, conducted data analysis, and wrote up the manuscript. EJ assisted with data collection and reviewed the manuscript. KA assisted in the write-up of the manuscript. EF assisted with data collection.

### Conflict of interest statement

The authors declare that the research was conducted in the absence of any commercial or financial relationships that could be construed as a potential conflict of interest.

## Footnotes

^1^Fulfilling these criteria is necessary before one can make credible group comparisons. However, it should be noted that there are other types of invariance (e.g., invariance of residuals; see Widaman and Reise, 1997) that, if lacking in a particular measure, may prohibit group comparisons.

## References

[B1] Ai A. L. (2004). Gender differences in war-related mental health issues among adult Kosovar refugees in the United States. J. Soc. Work. Res. 5, 81–97.

[B2] American Psychiatric Association (1987). Diagnostic and Statistical Manual of Mental Disorders, 3rd Edn. Washington, DC: American Psychiatric Association.

[B3] American Psychiatric Association (2013). Diagnostic and Statistical Manual of Mental Disorders, 5th Edn. Washington, DC: American Psychiatric Association.

[B4] Asparouhov T. Muthén B. (2014). Multiple-group factor analysis alignment. Struct. Equat. Model. 21, 495–508. 10.1080/10705511.2014.919210

[B5] Azocar F. Arean P. Miranda J. Munoz R. F. (2001). Differential item functioning in a Spanish translation of the Beck Depression Inventory. J. Clin. Psychol. 57, 355–365. 10.1002/jclp.1017 11241365

[B6] Bartholomew D. Knott M. Moustaki I. (2008). Latent Variable Models and Factor Analysis: A Unified Approach, 3rd Edn. New York, NY: Wiley.

[B7] Basishvili T. Eliozishvili M. Maisuradze L. Lortkipanidze N. Nachkebia N. Oniani T. (2012). Insomnia in a displaced population is related to war-associated remembered stress. Stress Health 28, 186–192. 10.1002/smi.1421 22282401

[B8] Bass J. K. Bolton P. A. Murray L. K. (2007). Do not forget culture when studying mental health. Lancet 370, 918–919. 10.1016/S0140-6736(07)61426-3 17869621

[B9] Beck A. T. Steer R. A. (1987). Manual for Revised Beck Depression Inventory. San Antonio, TX: Psychological Corporation.

[B10] Beck A. T. Steer R. A. (1993). Beck Depression Inventory: Manual. San Antonio, TX: Psychological Corp.

[B11] Beck A. T. Steer R. A. Brown G. K. (1996). Manual for the Beck Depression Inventory-2. San Antonio, TX: Psychological Corporation.

[B12] Beck A. T. Steer R. A. Garbin M. G. (1988). Psychometric properties of the Beck Depression Inventory: twenty-five years of evaluation. Clin. Psychol. Rev. 8, 77–100. 10.1016/0272-7358(88)90050-5

[B13] Bhui K. Abdi A. Abdi M. Pereira S. Dualeh M. Robertson D.. (2003). Traumatic events, migration characteristics and psychiatric symptoms among Somali refugees: preliminary communication. Soc. Psychiatry Psychiatr. Epidemiol. 38, 35–43. 10.1007/s00127-003-0596-5 12563557

[B14] Brouwer D. Meijer R. R. Zevalkink J. (2013). On the factor structure of the Beck Depression Inventory-II: G is the key. Psychol. Assess. 25, 136–145. 10.1037/a0029228 22800089

[B15] Brown T. H. Mellman T. A. (2014). The influence of PTSD, sleep fears, and neighborhood stress on insomnia and short sleep duration in urban, young adult, African Americans. Behav. Sleep Med. 12, 198–206. 10.1080/15402002.2013.784704 23767868 PMC3966964

[B16] Browne M. W. (2001). An overview of analytic rotation in exploratory factor analysis. Multivariate Behav. Res. 36, 111–150. 10.1207/S15327906MBR3601_05

[B17] Chen F. F. (2008). What happens if we compare chopsticks with forks? The impact of making inappropriate comparisons in cross-cultural research. J. Pers. Soc. Psychol. 95, 1005–1018. 10.1037/a0013193 18954190

[B18] Chentsova-Dutton Y. Tsai J. L. (2002). Understanding depression across cultures, in Handbook of Depression, eds Gotlib I. H. Hammen C. L. (New York, NY: Guilford Press), 467–491.

[B19] Delisle V. C. Beck A. T. Ziegelstein R. C. Thombs B. D. (2012). Symptoms of heart disease or its treatment may increase Beck Depression Inventory scores in hospitalized post-myocardial infarction patients. J. Psychosom. Res. 73, 157–162. 10.1016/j.jpsychores.2012.07.001 22850253

[B20] DeMars C. E. (2013). A tutorial on interpreting bifactor model scores. Int. J. Test. 13, 354–378. 10.1080/15305058.2013.799067

[B21] de Miranda Azevedo R. Roest A. M. Carney R. M. Denollet J. Freedland K. E. Grace S. L.. (2016). A bifactor model of the Beck Depression Inventory and its association with medical prognosis after myocardial infarction. Health Psychol. 35, 614–624. 10.1037/hea0000316 26901082

[B22] Dere J. Watters C. A. Yu S. C.-M. Bagby R. M. Ryder A. G. Harkness K. L. (2015). Cross-cultural examination of measurement invariance of the Beck Depression Inventory-II. Psychol. Assess. 27, 68–81. 10.1037/pas0000026 25314096

[B23] Farhood L. F. Dimassi H. (2012). Prevalence and predictors for post-traumatic stress disorder, depression and general health in a population from six villages in South Lebanon. Soc. Psychiatry Psychiatr. Epidemiol. 47, 639–649. 10.1007/s00127-011-0368-6 21455787

[B24] Fazel M. Wheeler J. Danesh J. (2005). Prevalence of serious mental disorder in 7000 refugees resettled in western countries: a systematic review. Lancet 365, 1309–1314. 10.1016/S0140-6736(05)61027-6 15823380

[B25] Fischer R. Fontaine J. R. J. (2011). Methods for investigating structural equivalence, in Cross-Cultural Research Methods in Psychology, eds Matsumoto D. Van de Vijver F. J. R. (New York, NY: Cambridge University Press), 179–215.

[B26] Foa E. B. Hembree E. A. Cahill S. P. Rauch S. A. M. Riggs D. S. Feeny N. C.. (2005). Randomized trial of prolonged exposure for posttraumatic stress disorder with and without cognitive restructuring: outcome at academic and community clinics. J. Consult. Clin. Psychol. 73, 953–964. 10.1037/0022-006X.73.5.953 16287395

[B27] Ghazinour M. Richter J. Eisemann M. (2003). Personality related to coping and social support among Iranian refugees in Sweden. J. Nerv. Ment. Dis. 191, 595–603. 10.1097/01.nmd.0000087186.03513.38 14504569

[B28] Gillespie C. F. Bradley B. Mercer K. Smith A. K. Conneely K. Gapen M.. (2009). Trauma exposure and stress-related disorders in inner city primary care patients. Gen. Hosp. Psychiatry 31, 505–514. 10.1016/j.genhosppsych.2009.05.003 19892208 PMC2785858

[B29] Hambrick J. P. Rodebaugh T. L. Balsis S. Woods C. M. Mendez J. L. (2010). Cross-ethnic measurement equivalence of depression, social anxiety, and worry. Assessment 17, 155–171. 10.1177/1073191109350158 19915199

[B30] Haroz E. E. Bolton P. Gross A. Chan K. S. Michalopoulos L. Bass J. (2016). Depression symptoms across cultures: an IRT analysis of standard depression symptoms using data from eight countries. Soc. Psychiatry Psychiatr. Epidemiol. 51, 981–991. 10.1007/s00127-016-1218-3 27083900 PMC6022281

[B31] Hollifield M. Warner T. D. Lian N. Krakow B. Jenkins J. H. Kesler J.. (2002). Measuring trauma and health status in refugees: a critical review. JAMA 288, 611–621. 10.1001/jama.288.5.611 12150673

[B32] Husain F. Anderson M. Cardozo B. L. Becknell K. Blanton C. Araki D. (2011). Prevalence of war-related mental health conditions and associations with displacement status in postwar Jaffna district, Sri Lanka. JAMA 306, 522–531. 10.1001/jama.2011.1052 21813430

[B33] Jayawickreme E. Jayawickreme N. Goonasekera M. A. (2012). Using focus group methodology to adapt measurement scales and explore questions of wellbeing and mental health: the case of Sri Lanka. Intervention 10, 156–167. 10.1097/WTF.0b013e328356f3c4

[B34] Jayawickreme E. Jayawickreme N. Miller E. (2010). Triumphalism, fear and humiliation: the psychological legacy of Sri Lanka's civil war. Dyn. Asymm. Confl. 3, 208–222. 10.1080/17467586.2010.531031

[B35] Jayawickreme N. Cahill S. P. Riggs D. S. Rauch S. A. M. Resick P. A. Rothbaum B. O.. (2014). Primum non nocere (first do no harm): symptom worsening and improvement in female assault victims after prolonged exposure for PTSD. Depress. Anxiety 31, 412–419. 10.1002/da.22225 24382682

[B36] Jayawickreme N. Jayawickreme E. Atanasov P. Goonasekera M. A. Foa E. B. (2012). Are culturally specific measures of trauma-related anxiety and depression needed? The Case of Sri Lanka. Psychol. Assess. 24, 791–800. 10.1037/a0027564 22429206

[B37] Jayawickreme N. Jayawickreme E. Goonasekera M. A. Foa E. B. (2009). Distress, wellbeing and war: qualitative analyses of civilian interviews from north-eastern Sri Lanka. Intervention 7, 204–222. 10.1097/WTF.0b013e328334636f

[B38] Jennrich R. I. Bentler P. M. (2011). Exploratory bi-factor analysis. Psychometrika 76, 537–549. 10.1007/s11336-011-9218-4 22232562 PMC3253271

[B39] Jennrich R. I. Bentler P. M. (2012). Exploratory bi-factor analysis: the oblique case. Psychometrika 77, 442–454. 10.1007/s11336-012-9269-1 27519775

[B40] Karasz A. (2005). Cultural differences in conceptual models of depression. Soc. Sci. Med. 60, 1625–1635. 10.1016/j.socscimed.2004.08.011 15652693

[B41] Kelly V. G. Merrill G. S. Shumway M. Alvidrez J. Boccellari A. (2010). Outreach, engagement, and practical assistance: essential aspects of PTSD care for urban victims of violent crime. Trauma Violence Abuse 11, 144–156. 10.1177/1524838010374481 20554505

[B42] Kessler R. C. Bromet E. J. (2013). The epidemiology of depression across cultures. Annu. Rev. Public Health 34, 119–138. 10.1146/annurev-publhealth-031912-114409 23514317 PMC4100461

[B43] Khoury L. Tang Y. L. Bradley B. Cubells J. F. Ressler K. J. (2010). Substance use, childhood traumatic experience, and posttraumatic stress disorder in an urban civilian population. Depress. Anxiety 27, 1077–1086. 10.1002/da.20751 21049532 PMC3051362

[B44] Killgore W. D. S. (1999). Empirically derived factor indices for the Beck Depression Inventory. Psychol. Rep. 84, 1005–1013. 10.2466/pr0.1999.84.3.1005 10408220

[B45] Kirmayer L. J. Young A. (1998). Culture and somatization: clinical, epidemiological and ethnographic perspectives. Psychosom. Med. 60, 420–430. 10.1097/00006842-199807000-00006 9710287

[B46] Kline R. B. (2016). Principles and Practice of Structural Equation Modeling, 4th Edn. New York, NY: Guilford Press.

[B47] Kuittinen S. Punamäki R.-L. Mölsä Saarni S. I. Tiilikainen M. Honkasalo M.-L. (2014). Depressive symptoms and their psychosocial correlated among older Somali Refugees and Native Finns. J. Cross-Cult. Psychol. 45, 1434–1452. 10.1177/0022022114543519

[B48] Lahlou-Laforet K. Ledru F. Niarra R. Consoli S. M. for the PANIC investigators (2015). Validity of Beck Depression Inventory for the assessment of depressive mood in chronic heart failure patients. J. Affect. Disord. 184, 256–260. 10.1016/j.jad.2015.05.056 26118753

[B49] Li C.-H. (2016). The performance of ML, DWLS, and ULS estimation with robust corrections in structural equation models with ordinal variables. Psychol. Methods 21, 369–387. 10.1037/met0000093 27571021

[B50] Lopez S. R. Guarnaccia P. J. J. (2000). Cultural psychopathology: uncovering the social world of mental illness. Annu. Rev. Psychol. 51, 571–598. 10.1146/annurev.psych.51.1.571 10751981

[B51] Lorenzo-Seva U. ten Berge J. M. F. (2006). Tucker's congruence coefficient as a meaningful index of factor similarity. Methodology 2, 57–64. 10.1027/1614-2241.2.2.57

[B52] Luitel N. P. Jordans M. J. D. Sapkota R. P. Tol W. A. Kohrt B. A. Thapa S. B.. (2013). Conflict and mental health: a cross-sectional epidemiological study in Nepal. Soc. Psychiatry Psychiatr. Epidemiol. 48, 183–193. 10.1007/s00127-012-0539-0 22777395

[B53] MacCallum R. C. Widaman K. F. Zhang S. Hong S. (1999). Sample size in factor analysis. Psychol. Methods 4, 84–99. 10.1037/1082-989X.4.1.84

[B54] Magruder K. M. Kiliç C. Koryürek M. M. (2015). Relationship of posttraumatic growth to symptoms of posttraumatic stress disorder and depression: a pilot study of Iraqi students. Int. J. Psychol. 50, 402–406. 10.1002/ijop.12146 25691475

[B55] Mansolf M. Reise S. P. (2016). Exploratory bifactor analysis: the Schmid-Leiman orthogonalization and Jennrich-Bentler analytic rotations. Multivariate Behav. Res. 51, 698–717. 10.1080/00273171.2016.1215898 27612521 PMC5425103

[B56] McDonald R. P. (1999). Test Theory: A Unified Treatment. Mahwah, NJ: Lawrence Erlebaum & Associates.

[B57] Mghir R. Raskin A. (1999). The psychological effects of the war in Afghanistan on young Afghan refugees from different ethnic backgrounds. Int. J. Soc. Psychiatry 45, 29–40. 10.1177/002076409904500104 10443247

[B58] Millsap R. E. (2011). Statistical Approaches to Measurement Invariance. New York, NY: Routledge.

[B59] Missinne S. Vandeviver C. Van de Velde S. Bracke P. (2014). Measurement equivalence of the CES-D depression-scale among the ageing population in eleven European countries. Soc. Sci. Res. 46, 38–47. 10.1016/j.ssresearch.2014.02.006 24767588

[B60] Mölsä M. Punamaki R.-L. Saarni S. I. Tiilikainen M. Kuittinen S. Honkasalo M.-L. (2014). Mental and somatic health and pre- and post-migration factors among older Somali refugees in Finland. Transcult. Psychiatry 51, 499–525. 10.1177/1363461514526630 24648488

[B61] Muthén L. K. Muthén B. O. (1998–2015). Mplus User's Guide, Seventh Edition. Los Angeles, CA: Muthén & Muthén.

[B62] Nuevo R. Dunn G. Dowrick C. Vázquez-Barquero J. L. Casey P. Dalgard O. S.. (2009). Cross-cultural equivalence of the Beck Depression Inventory: a five-country analysis from the ODIN study. J. Affect. Disord. 114, 156–162. 10.1016/j.jad.2008.06.021 18684511

[B63] Paniagua F. A. (2000). Culture-bound syndromes, cultural variations, and psychopathology, in Handbook of Multicultural Mental Health: Assessment and Treatment of Diverse Populations, eds Cuellar I. Paniagua F. A. (San Diego, CA: Academic), 139–169.

[B64] Post L. M. Zoellner L. A. Youngstrom E. Feeny N. C. (2011). Understanding the relationship between co-occurring PTSD and MDD: symptom severity and affect. J. Anxiety Disord. 25, 1123–1130. 10.1016/j.janxdis.2011.08.003 21899984 PMC3196268

[B65] Radloff L. (1977). The CES-D scale: a self-report depression scale for research in the general population. Appl. Psychol. Meas. 1, 385–401. 10.1177/014662167700100306

[B66] Rasmussen A. Verkuilen J. Ho E. Fan Y. (2015). Posttraumatic stress disorder among refugees: measurement invariance of Harvard Trauma Questionnaire scores across global regions and response patterns. Psychol. Assess. 27, 1160–1170. 10.1037/pas0000115 25894706 PMC4615261

[B67] Reise S. P. (2012). Invited paper: the rediscovery of bifactor measurement models. Multivariate Behav. Res. 47, 667–696. 10.1080/00273171.2012.715555 24049214 PMC3773879

[B68] Renner W. Salem I. (2009). Post-traumatic stress in asylum seekers and refugees from Chechnya, Afghanistan, and West Africa: gender differences in symptomatology and coping. Int. J. Soc. Psychiatry 55, 99–108. 10.1177/0020764008092341 19240200

[B69] Richter P. Werner J. Heerlein A. Kraus A. Sauer H. (1998). On the validity of the Beck Depression Inventory. Psychopathology 31, 160–168. 10.1159/000066239 9636945

[B70] Rohlof H. G. Knipscheer J. W. Kleber R. J. (2014). Somatization in refugees: a review. Soc. Psychiatry Psychiatr. Epidemiol. 49, 1793–1804. 10.1007/s00127-014-0877-1 24816685

[B71] Rothbaum B. O. Foa E. B. Riggs D. S. Murdock T. Walsh W. (1992). A prospective examination of post-traumatic stress disorder in rape victims. J. Trauma Stress 5, 455–475. 10.1002/jts.2490050309

[B72] Ryder A. G. Yang J. Zhu X. Yao S. Heine S. J. Bagby R. M. (2008). The cultural shaping of depression: somatic symptoms in China, psychological symptoms in North America? J. Abnorm. Psychol. 117, 300–313. 10.1037/0021-843X.117.2.300 18489206

[B73] Saxena S. Thornicroft G. Knapp M. Whiteford H. (2007). Resources for mental health: scarcity, inequity, and inefficiency. Lancet 370, 878–889. 10.1016/S0140-6736(07)61239-2 17804062

[B74] Seglem K. B. Oppedal B. Raeder S. (2011). Predictors of depressive symptoms among resettled, unaccompanied refugee minors. Scand. J. Psychol. 52, 457–464. 10.1111/j.1467-9450.2011.00883.x 21895671

[B75] Shafer A. B. (2006). Meta-analysis of the factor structures of four depression questionnaires: Beck, CES-D, Hamilton, and Zung. J. Clin. Psychol. 62, 123–146. 10.1002/jclp.20213 16287149

[B76] Song S. J. Kaplan C. Tol W. A. Subica A. de Jong J. (2015). Psychological distress in torture survivors: pre- and post-migration risk factors in a US sample. Soc. Psychiatry Psychiatr. Epidemiol. 50, 549–560. 10.1007/s00127-014-0982-1 25403567

[B77] Steel Z. Chey T. Silove D. Marnane C. Bryant R. A. van Ommeren M. (2009). Association of torture and other potentially traumatic events with mental health outcomes among populations exposed to mass conflict and displacement: a systematic review and meta-analysis. JAMA 302, 537–549. 10.1001/jama.2009.1132 19654388

[B78] Steer R. A. Iguchi M. Y. Platt J. J. (1992). Use of the revised Beck Depression Inventory with intravenous drug users not in treatment. Psychol. Addict. Behav. 6, 225–232. 10.1037/h0080631

[B79] Suija K. Rajala U. Jokelainen J. Liukkonen T. Harkonen P. Keinanen-Kiukaanniemi S.. (2012). Validation of the Whooley questions and the Beck Depression Inventory in older adults. Scan. J. Prim. Health Care 30, 259–264. 10.3109/02813432.2012.732473 23113732 PMC3520422

[B80] Udo T. McKee S. A. Grilo C. M. (2015). Factor structure and clinical utility of the Beck Depression Inventory in patients with binge eating disorder and obesity. Gen. Hosp. Psychiatry 37, 120–125. 10.1016/j.genhosppsych.2014.11.011 25537344 PMC4361288

[B81] United Nations High Commissioner for Refugees (2016). Global Trends: Forced Displacement in 2015. Geneva: United Nations High Commissioner for Refugees.

[B82] Van Hemert D. A. Van De Vijver F. J. R. Poortinga Y. H. (2002). The Beck Depression Inventory as a measure of subjective well-being: a cross-national study. J. Happiness Stud. 3, 257–286. 10.1023/A:1020601806080

[B83] Van Ommeren M. (2003). Validity issues in transcultural epidemiology. Br. J. Psychiatry 182, 376–378. 10.1192/bjp.182.5.376 12724237

[B84] Van Ommeren M. Sharma B. Thapa S. Makaju R. Prasain D. Bhattarai R. (1999). Preparing instruments for transcultural research: use of the Translation Monitoring Form with Nepali-speaking Bhutanese Refugees. Transcult. Psychiatry 36, 285–301. 10.1177/136346159903600304

[B85] Veerman J. L. Dowrick C. Ayuso-Mateos J. L. Dunn G. Barendregt J. J. (2009). Population prevalence of depression and mean Beck Depression Inventory score. Br. J. Psychiatry 195, 516–519. 10.1192/bjp.bp.109.066191 19949201

[B86] Ward L. C. (2006). Comparison of factor structure models for the Beck Depression Inventory-II. Psychol. Assess. 18, 81–88. 10.1037/1040-3590.18.1.81 16594815

[B87] Wardenaar K. J. Wanders R. B. K. Roest A. M. Meijer R. R. De Jonge P. (2015). What does the Beck Depression Inventory measure in myocardial infarction patients? A psychometric approach using item response theory and person-fit. Int. J. Methods Psychiatr. Res. 24, 130–142. 10.1002/mpr.1467 25994207 PMC6878327

[B88] Whisman M. A. Judd C. M. Whiteford N. T. Gelhorn H. L. (2012). Measurement invariance of the Beck Depression Inventory – Second Edition (BDI-II) across gender, race, ethnicity in college students. Assessment 20, 419–428. 10.1177/1073191112460273 23044895

[B89] Whitbeck L. B. Armenta B. E. Gentzler K. C. (2015). Homelessness-related traumatic events and PTSD among women experiencing episodes of homelessness in three U.S. cities. J. Trauma Stress 28, 355–360. 10.1002/jts.22024 26184885

[B90] Whiteford H. A. Ferrari A. J. Dengenhardt L. Feigin V. Vos T. (2015). The global burden of mental, neurological, and substance use disorders: an analysis from the Global Burden of Disease Study 2010. PLoS ONE 10:e0116820. 10.1371/journal.pone.0116820 25658103 PMC4320057

[B91] Widaman K. F. Reise S. P. (1997). Exploring the measurement invariance of psychological instruments: applications in the substance use domain, in The Science of Prevention: Methodological Advances from Alcohol and Substance Abuse Research, eds Bryant K. J. Windle M. E. West S. G. (Washington, DC: American Psychological Association), 281–324.

[B92] Yurtbay T. Alyanak B. Abali O. Kaynak N. Durukan M. (2003). The psychological effects of forced emigration on Muslim Albanian children and adolescents. Community Ment. Health 39, 203–212. 10.1023/A:1023386122344 12836802

